# Quality of life data for individuals affected by spinal muscular atrophy: a baseline dataset from the Cure SMA Community Update Survey

**DOI:** 10.1186/s13023-020-01498-2

**Published:** 2020-08-24

**Authors:** Lisa Belter, Rosángel Cruz, Jill Jarecki

**Affiliations:** Cure SMA, Elk Grove Village, IL USA

**Keywords:** Spinal muscular atrophy, Health utilities index, Work productivity and activity impairment, PROMIS fatigue short form, Patient reported outcome measures, Health related quality of life

## Abstract

**Background:**

Individuals and/or caregivers of individuals affected by spinal muscular atrophy (SMA) completed the 2019 Cure SMA Community Update Survey, online, assessing health-related quality of life (HRQoL), loss of work productivity, and fatigue using the Health Utilities Index Questionnaire (HUI), the Work Productivity and Activity Impairment Questionnaire (WPAI), and the Patient Reported Outcomes Measurement Information System Fatigue Short Form (PROMIS Fatigue SF), respectively. The purpose was to collect baseline quality of life results among individuals affected by SMA using the above Patient Reported Outcome Measures (PROMs).

**Results:**

Of 666 surveys completed between March and May 2019, 478 were included in this analysis, accounting for duplicates, missing data, or deaths. The breakdown across SMA type I, II and III was 25, 47 and 28%, respectively. Responses were characterized by current functional status/milestone, with subsets for “permanent ventilation,” “non-sitters,” “sitters,” “walk with support,” and “walk alone.” WPAI and HUI respondents included affected adults and caregivers. The PROMIS Fatigue SF was completed by the primary caregiver of affected children.

Overall, those affected by a less severe form of SMA and with a higher functional status reported higher HRQoL and lower work productivity and activity impairment. All affected individuals reported higher fatigue levels than the general population.

**Conclusions:**

This study offers useful insights into the burden of SMA among affected individuals and their caregivers. The results provide a baseline picture of the patient and caregiver experience with SMA in a post-treatment era from which to measure year-over-year changes in quality of life scores from new therapies and improved care. The WPAI demonstrates the significant impact of work productivity among SMA populations. Aspects of the HUI seem more appropriate to certain SMA sub-populations than others. Measures from the PROMIS Fatigue SF appear to under-represent the burden of fatigue often reported by SMA individuals and caregivers; this may, perhaps be due to a lack of sensitivity in the questions associated with fatigue in the SMA affected population, when compared with other studies on this topic. Overall, these results suggest the need for SMA-specific quality of life outcome measures to fully capture clinically meaningful change in the SMA population.

## Background

Spinal muscular atrophy (SMA) is a genetic neuromuscular disease characterized by progressive muscle atrophy and weakness that historically often led to paralysis and premature death [1]. While SMA most often first presents among infants and toddlers, it also can present in juveniles and, less frequently in adults. SMA has traditionally been classified into clinical subtypes based upon age of onset and the highest physical milestone achieved [2].

Type I SMA (Werdnig Hoffman Disease) presents with severe generalized weakness, hypotonia, tongue fasciculations, and impaired respiratory function, difficulty feeding and swallowing, among other symptoms appearing within the first 6 months of life. In the absence of treatment, most of these infants required intensive supportive care, as they were never able to sit, and lost their ability to breathe and feed independently. Historically, more than half of children with type I SMA did not survive to age two [3]. This is changing with new FDA approved therapies [4, 5]. Type II SMA (Kugelberg Welander disease) usually presents between the ages of 6 and 18 months, with affected children being able to sit independently but unable to walk without assistance, with most experiencing respiratory difficulties and progressive scoliosis, and some developing eventual problems with chewing and swallowing. Patients with juvenile SMA (type III) can walk at some point and have a normal life expectancy, although they develop muscle weakness over time and often eventually lose ambulation. In rare cases, patients’ symptoms first appear in adulthood (type IV) [6–8].

As mentioned, the SMA treatment landscape changed dramatically in late 2016 with FDA approval of nusinersen, the first disease modifying therapy for SMA, and then with the FDA approval of onasemnogene abeparvovec-xioi in 2019, demonstrating that it is possible to impact rates of survival, motor function, and respiratory function for SMA patients [5, 9, 10]. Access to these novel therapies coupled with improvements in supportive care are not only rapidly changing the therapeutic landscape but also the quality of life of those affected and how they may experience SMA [5, 10, 11]. Despite this progress, there is limited qualitative and quantitative data that have been collected on the burden of SMA, particularly after the approval of these treatments.

What we have learned through qualitative research conducted prior to approval of new treatment options, is that the burden of SMA is multifaceted and extremely challenging for patients and their families. Often this odyssey begins with a prolonged and traumatic process to confirm diagnosis and a life-long journey of overwhelming physical, emotional, psychosocial, and financial strains associated with managing and living with a progressive, debilitating, and incurable disease [7, 12–15].

As we continue to capture and optimize care and treatment access in SMA, it is critical to quantify outcomes that are meaningful from the patient perspective, and which measure the impact of therapies on other dimensions of life other than assessing survival or significant changes in motor milestones. For instance, the Institute for Clinical and Economic Review (ICER) published a report, in April 2019, on the FDA-approved treatments called Spinraza® and Zolgensma® for Spinal Muscular Atrophy: Effectiveness and Value [16]. The original ICER report found that neither medication met the standard quality-adjusted life year (QALY) cost-effectiveness threshold nor did they meet the modified QALY threshold for rare diseases, even though both drugs are transformative when given early.

Importantly, it has also been suggested by the patients themselves, as well as healthcare providers, that the factors being assessed in such pharmacoeconomic analyses are not sensitive enough to capture the overall impact on quality of life [17]. Thus, it has been proposed for SMA (and for rare disease more generally) that focusing on generating evidence that translates therapy benefit from clinical trials to patient and family relevant outcomes such as quality of life, independence and productivity impact might be more appropriate when analyzing cost effectiveness. Encompassing these additional dimensions would provide a more complete picture of the burden of disease and the potential overall impact of a new therapy [18]. With similar efforts recently undertaken in other rare, pediatric diseases such as Duchenne Muscular Dystrophy (DMD), there has been strong interest in collecting health utility and HRQoL measures for the SMA population [19].

This study looks at various PROMs that assess specific aspects of the patient experience that might be more sensitive in capturing subtle, but meaningful changes with drug therapy. Leveraging its annual online Community Update Survey [20], in 2019 Cure SMA engaged respondents in completing three commonly used PROMs: the Health Utilities Index (HUI), Work Productivity and Activity Impairment (WPAI) and the Patient Reported Outcomes Measurement Information System Fatigue Short Form (PROMIS Fatigue SF).

The study assesses various PROMs that address specific aspects of the patient experience to determine whether the PROMs are sensitive in capturing subtle, but meaningful changes with drug therapy administration in order to benchmark the progression of SMA, and other aspects of disease impact such as quality of life, work productivity, and fatigue. The authors also present a discussion of the strengths and limitations of these three PROM tools when used in the SMA setting, suggesting the need for future efforts to develop and validate SMA-specific outcome measures.

## Methods

### Participants

The Cure SMA database members were the primary target population for these surveys. Cure SMA is the largest SMA patient advocacy organization based in the US and maintains the largest self-reported database on individuals with SMA worldwide [21]. More specifically, the patient-reported database contains self-reported records for more than 8000 affected individuals, including information relating to demographics, SMA type, and diagnosis date [21]. Beginning in 2017, the organization launched an annual online Community Update Survey of this database to more comprehensively capture the natural history of SMA, from the patient’s perspective and to develop additional data that can support assessment of the impact of SMA. In the 2019 survey participants were asked to complete information on a range of topics including demographic information (e.g., sex, age at survey, vital status, educational level, employment); clinical disease characteristics (e.g., age at diagnosis and symptom onset, SMN2 gene copy number) and family history; respiratory interventions, motor function, surgeries and hospitalizations; and participation in clinical trials and treatment. Additionally, the survey included a specific assessment of HRQoL, work productivity and activity impairment, and fatigue. To minimize survey fatigue among a chronically affected patient community, only three assessments covering HRQoL, work productivity and activity impairment were used in the 2019 survey. Details on a previously used assessment in the 2017 Community Update Survey, the PedsQL™ [22], was presented at the 22nd International Congress of the World Muscle Society [23]. Respondents were stratified within the final dataset by the affected individual’s SMA type and current functional status. Functional status was categorized as requiring permanent ventilation (defined as 16 or more hours per day of breathing support); non-sitters (defined as lacking head control, voluntary grasping, voluntary kicking or roll-over completely and not requiring permanent ventilation); sitters (defined as able to sit without support, stand alone or stand with assistance); walk with support; and walk independently. These states were treated as mutually exclusive and identified from the survey responses.

Over 4000 survey invitations were sent out to both parents of affected children and affected adults within the Cure SMA database in March 2019 via email and mailed postcards. It was also posted to the Cure SMA website and social media sites. Surveys were accepted from both US and international families. IRB approval was obtained by Western IRB (IRB Report ID: 1785926). All data were de-identified prior to analysis.

### Outcome measures & statistical approach

#### Health utilities index

To assess overall health-related quality of life utility scores within the SMA population, Cure SMA used the Health Utilities Index Mark 3 (HUI3) system as its tool [24]. It defines 972,000 unique health states. This classification system provides a comprehensive framework within which to describe health status and develop assessment of health-related quality of life (HRQL) and has been used successfully in rare disease states impacting children [19] ages 5 and up, but has not yet been validated in an SMA population. Health-related quality of life (HRQoL), has been defined as “the value assigned to duration of life as modified by the impairments, functional states, perceptions, and social opportunities that are influenced by disease, injury, treatment, or policy [25].” Although there are other systems available within the HUI, such as the HUI Mark 1 (HUI1) and the HUI Mark 2 (HUI2) only the HUI3 results are included in this analysis. The HUI1 was originally developed to assess the health of children who had been in neonatal intensive care and is now seldom used [24]. The HUI2 assesses seven attributes of quality of life: vision, hearing, speech, mobility, emotion, cognition, self-care, pain and fertility. The HUI3 assess eight attributes of quality of life: vision, hearing, speech, ambulation, dexterity, emotion, cognition and pain. Both the HUI2 and HUI3 results were computed following the survey, but given the HUI3 provided more discrimination across functional status than the HUI2 and to prevent redundancy of results, Cure SMA concluded that only the HUI3 data will be presented here as it provided the more detailed and relevant descriptive system, full structural independence and population norms most relevant to SMA. This additional baseline data for the SMA population can be used in secondary analyses and future comparative studies.

For this analysis, a single global utility score was developed for sub-types based on functional milestone to isolate health quality of life experience among the relevant sub-populations. HUI scores are calculated using the HUI3 health status classification system and utility scoring function. HUI scores range from − 0.36 (worst possible health state) through 0.00 (death) to 1.00 (perfect health). Alternative to the continuous HUI3 utility scores, is to group them into disability categories, such that a score of 1.00 represents perfect health, scores of 0.89–0.99 represent mild disability, scores of 0.70–0.88 represent moderate disability, and scores less than 0.70 represent severe disability [26]. Additionally, the scores of the eight single attributes of quality of life as defined by the HUI3 health status classification system (vision, hearing, speech, ambulation, dexterity, emotion, cognition, and pain) are also presented here by SMA type. The scores range from 0.00 (death) to 1.00 (perfect health) [24]. Due to the non-normal distribution of the attribute scores by SMA type, the Kruskal-Wallis H test was used to determine statistically significant differences between attribute scores by type. The HUI was completed by parents of affected children ages 5 and up and affected adults ages 18 and up. Children did not complete the HUI.

#### Work productivity and activity impairment questionnaire

Cure SMA selected the Work Productivity and Activity Impairment Questionnaire (WPAI) [27] to assess impact of SMA on productivity for both affected adults and caregivers of affected children. The WPAI is a six-question survey assessing the effect of a problem on an individual’s ability to work and perform regular activities with the prior 7 days. The WPAI has been validated for use in a variety of disease states and has been used in SMA clinical trials [28] but not validated in an SMA population. The assessment tool yields scores on work time missed (absenteeism), impaired productivity at work (presenteeism), overall work productivity loss (absenteeism and presenteeism combined) and impairment in non-work-related activities due to health problems (activity impairment). WPAI outcomes are expressed as impairment percentages, with higher numbers indicating greater impairment and less productivity. Independent t-tests were used to test for statistically significant differences in work productivity scores of a caregiver verses an affected adult to determine if work productivity was impacted differently among those caring for someone with SMA versus someone affected with SMA. Where noted, as required Mann-Whitney U tests were used for testing differences in work productivity scores with non-normal distributions. A linear regression was used to determine if work productivity was impacted by the age of the affected individual. A linear regression was chosen because both the independent variables (WPAI sub-scores) and dependent variable (age during survey) were continuous variables.

#### PROMIS fatigue SF

Fatigue is a common concern among patients with SMA [29]. Fatigue can be described by two domains: an objective one, also referred to as fatigability (susceptibility to decline in motor performance due to weakness or loss of strength) and a subjective one, also referred to as perceived fatigue (which consists of the sense of tiredness or lack of energy) [30, 31]. While fatigability is commonly evaluated during clinical assessments, perceived fatigue has only begun to be studied in the SMA population. To assess perceived fatigue, Cure SMA selected the PROMIS Fatigue SF parent proxy survey instrument [32] because of its available parent proxy version, limited set of questions and free public access. This tool measures the experience and impact of fatigue among various populations but has not yet been validated in an SMA population. Fatigue is measured over the prior 7 days using a 10-item assessment tool. Higher scores indicate greater fatigue. For the SMA survey, the form was completed by parents of affected children between the ages of 5 and 17. For this analysis, T-scores were developed to rescale the raw score into a standardized score with a mean of 50 and a standard deviation of 10. Scoring was stratified by SMA type and functional milestone and compared to the general US population. Due to the small sample sizes of each subpopulation, statistical significance testing was not done. However, to explore further which question(s) among the PROMIS Fatigue SF may not be sensitive or appropriate to use in the SMA population, a separate factor analysis was carried out using a one-way ANOVA statistical test to assess the relationship between each question of the PROMIS Fatigue SF by SMA type and functional status.

## Results

### Demographics

**Fig. 1:**
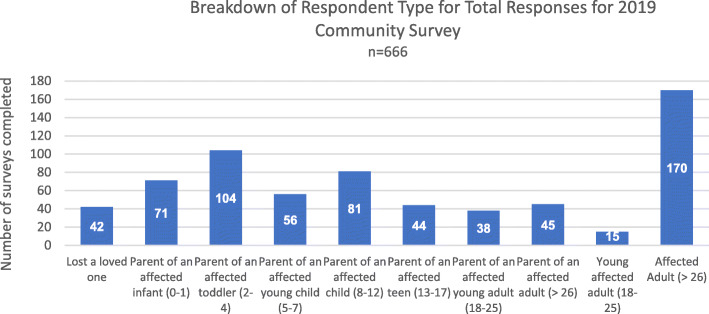
Breakdown of Respondent Type for Total Responses for 2019 Community Survey

In total, 666 responses representing 639 unique individuals were received between March and May 2019, with the majority, 481, filled out on behalf of the affected individual by a parent/caregiver and 185 filled out directly by an affected individual (18 years of age and older) (Fig. 1).

Thirty-four duplicate surveys completed for the same affected individual (i.e.: both parents submitted surveys), 63 surveys missing functional status information, 20 surveys relating to deceased individuals, 55 surveys missing diagnosis date and 16 surveys with SMA type other than I, II, or III were excluded from the final dataset. A total of 478 records, 121 representing type I (25%), 225 representing type II (47%) and 132 representing type III (28%), remained, but not every assessment was completed by all survey participants because of age and type of respondent. The final dataset included 194 affected males and 284 affected females, with an average age of just over 17 years. Survey respondents had a mean age at symptom onset of 2.6, 10.7, and 33.9 months for types I, II, and III respectively, and had a mean age of diagnosis at 4.0, 24.6, and 110.4 months (Table 1).

**Fig. 2:**
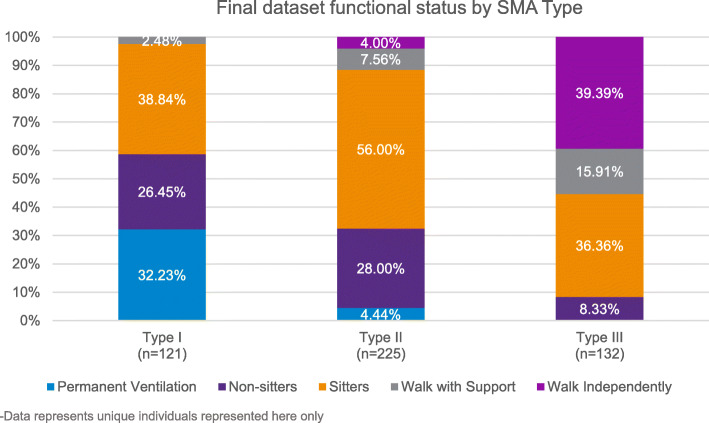
Final dataset functional status by SMA Type

Among those representing type I affected individuals, 32.23% required permanent ventilation, 26.45% were non-sitters but did not require permanent ventilation, 38.84% were sitters, and 2.48% could walk with support. Among those representing type II affected individuals, 4.44% required permanent ventilation, 28% were non-sitters, 56% were sitters, 7.56% could walk with support and 4% could walk independently. Among those with type III SMA, 8.33% were non-sitters, 36.36% were sitters, 15.91% could walk with support and 39.39% could walk independently (Fig. 2). It is important to note that some of these atypical motor functions for their corresponding SMA type may have been due to response error, clinical trial participation and/or receiving a commercially approved therapy. That analysis is not shown here.

**Table 1:** Demographics of Affected Individual within Final Dataset for 2019 Community Survey Quality of Life Analysis

	Total	Type I	Type II	Type III
n (%)	478	121 (25.3)	225 (47.1)	132 (27.6)
Male, n (%)	194 (40.6)	54 (44.6)	86 (38.2)	54 (40.9)
Age at symptom onset in months, mean (SD)	15.1 (31.3)	2.6 (2.4)	10.7 (5.8)	33.9 (54.1)
Diagnostic delay in months^a^, mean (SD)	26.9 (67.9)	2.1 (2.3)	11.9 (30.3)	74.4 (108.5)
Age at diagnosis in months, mean (SD)	43.1 (83.2)	4.0 (4.4)	24.6 (39.5)	110.4 (126.2)
Age at time of survey in years, mean (SD)	17.1 (16.8)	4.1 (5.9)	17.2 (14.3)	28.6 (18.8)

Data represents unique individuals-only

*SD* standard deviation

^a^Diagnostic delay calculated for each individual by age at diagnosis (in months) minus age at symptom onset (in months)

### HUI3

Overall, the average HUI3 scores ranged from − 0.05 to 0.64 (Table 2). When looking at functional status with the HUI data, we observed that as functional status increased, the health utility rating also increased. For example, the average HUI3 score was 0.24 among those whose maximum motor function was sitting, and it increased to 0.64 for independent walkers. The mean HUI3 scores increased by increasing functional status and SMA type, with the lowest average HUI3 score among those with type I on permanent ventilation, − 0.05, and the highest average HUI3 score of 0.64 among those with type III that can walk independently. All scores regardless of SMA type and functional status remained in the severe disability category (less than 0.70) [26].

**Table 2:** HUI3 Scores by Functional Milestone and SMA Type

Functional Status	Type I	Type II	Type III
Permanent Ventilation			
n	18	9	0
Mean Score (SD)	− 0.05 (0.10)	0.10 (0.11)	
Range	(−0.20–0.13)	(− 0.02–0.31)	
Non-Sitters			
n	5	51	9
Mean Score (SD)	0.06 (0.10)	0.12 (0.12)	0.14 (0.13)
Range	(−0.03–0.21)	(− 0.16–0.41)	(− 0.10–0.34)
Sitters			
n	4	81	41
Mean Score (SD)	0.11 (0.21)	0.26 (0.16)	0.23 (0.11)
Range	(−0.14–0.35)	(−0.20–0.70)	(0.04–0.48)
Walk with Support			
n	0	6	13
Mean Score (SD)		0.44 (0.12)	0.35 (0.21)
Range		(0.32–0.59)	(0.02–0.63)
Walk Independently			
n	0	3	42
Mean Score (SD)		0.58 (0.15)	0.64 (0.24)
Range		(0.48–0.76)	(−0.04–1)

When examining the eight individual single-attribute HUI3 utility scores by SMA type, vision, hearing, and emotion (Table 3) had high utility scores (all above 0.9) across all three SMA types, representing none to mild disability. Additionally, there was no statistically significant differences in their utility scores across type. Ambulation was the attribute with the lowest utility scores, 0.01, 0.04, and 0.32, for type I, II, and III, respectively, representing severe disability, and there was a statistically significant difference in scores by type. The speech attribute utility score was 0.51 in SMA type I, representing severe disability, but the other SMA types scores, 0.95 and 0.99, for type II and type III, respectively, represented mild to severe disability. Additionally there, was a statistically significant difference by type among the speech attribute.

**Table 3:** HUI3 Attribute Scores by SMA Type

Attribute Scores	Type I	Type II	Type III	*p* value
Vision				
n	29	170	116	0.54
Mean Score (SD)	0.97 (0.05)	0.97 (0.06)	0.97 (0.03)	
Range	(0.73–1)	(0.59–1)	(0.95–1)	
Hearing				
n	30	167	118	0.60
Mean Score (SD)	0.99 (0.05)	1.0	0.99 (0.05)	
Range	(0.71–1)	(0.48–1)	(0.48–1)	
Speech				
n	31	168	118	0.0001*
Mean Score (SD)	0.51 (0.43)	0.95 (0.11)	0.99 (0.04)	
Range	(0–1)	(0.41–1)	(0.67–1)	
Ambulation				
n	31	171	122	0.0001*
Mean Score (SD)	0.01 (0.06)	0.04 (0.14)	0.32 (0.36)	
Range	(0–0.36)	(0–1)	(0–1)	
Dexterity				
n	31	168	120	0.0001*
Mean Score (SD)	0.13 (0.27)	0.57 (0.35)	0.78 (0.27)	
Range	(0–1)	(0–1)	(0–1)	
Emotion				
n	30	166	120	0.10
Mean Score (SD)	0.94 (0.14)	0.96 (0.08)	0.93 (0.13)	
Range	(0.33–1)	(0.33–1)	(0.33–1)	
Cognition				
n	30	167	118	0.001*
Mean Score (SD)	0.87 (0.25)	0.97 (0.09)	0.96 (0.11)	
Range	(0–1)	(0.32–1)	(0.32–1)	
Pain				
n	31	169	120	0.09
Mean Score (SD)	0.90 (0.11)	0.86 (0.16)	0.84 (0.16)	
Range	(0.48–1)	(0–1)	(0–1)	

*Statistically significant at *p* < 0.05

### WPAI

For the WPAI, sub-scores were stratified by caregiver and affected adults, and by functional milestone of individuals with SMA as reflected in the final dataset (Table 4). Results from the WPAI indicated that 47.95 and 22.01% of affected adults and caregivers, respectively, were currently employed (working for pay) at the time of the survey. Results on absenteeism, presenteeism, and overall impairment among affected individuals on permanent ventilation were not reported here due to small sample size (*n* < 2). There was a statistically significant difference in the percentage of work missed among caregivers of a child with SMA that could sit and an affected adult that could sit, with caregivers reporting higher percentage of work missed in the last 7 days. Additionally, there was a statistically significant difference in productivity lost in non-work related activities among caregivers of affected children that could walk independently and affected adults that could walk independently with higher productivity lost reported among the affected adults.

**Table 4:** WPAI Sub-scores Stratified by Caregiver and Affected Individual

Functional Status	Caregiver		Affected Adult		Overall		Significance testing^a^
	Mean (SD)	Sample Size	Mean (SD)	Sample Size	Mean (SD)	Sample Size	P value
Permanent Ventilation							
Absenteeism, %	7.6 (9.1)	4	N/A	N/A	6.1 (8.6)	5	N/A
Presenteeism, %	76.4 (31.1)	11	N/A	N/A	73.1 (34.7)	13	N/A
Overall Impairment, %	59.0 (43.3)	4	N/A	N/A	49.2 (43.4)	5	N/A
Activity Impairment, %	83.1 (20.9)	35	68.8 (25.3)	8	80.5 (22.2)	43	0.13^b^
Non-sitters							
Absenteeism, %	13.3 (15.1)	16	6.4 (13.6)	15	10.0 (14.6)	31	0.09^b^
Presenteeism, %	47.0 (31.8)	23	36.7 (29.5)	18	42.4 (30.9)	41	0.29
Overall Impairment, %	50.1 (27.3)	16	37.2 (28.8)	15	43.9 (28.3)	31	0.21
Activity Impairment, %	60.8 (29.4)	50	69.1 (21.5)	34	64.2 (26.7)	84	0.14
Sitters							
Absenteeism, %	18.8 (29.0)	36	6.8 (12.1)	31	13.3 (23.4)	67	0.04*
Presenteeism, %	41.6 (31.0)	45	44.5 (30.1)	40	42.9 (30.5)	85	0.66
Overall Impairment, %	44.6 (29.6)	33	41.0 (23.7)	30	42.9 (26.8)	63	0.59
Activity Impairment, %	60.9 (25.2)	123	55.2 (22.6)	65	58.9 (24.4)	188	0.09^b^
Walk with Support							
Absenteeism, %	16.3 (11.5)	6	8.5 (11.8)	4	13.2 (11.6)	10	0.34
Presenteeism, %	55.0 (31.2)	8	50.0 (33.7)	4	53.3 (30.6)	12	0.93^b^
Overall Impairment, %	62.7 (19.5)	6	53.2 (35.5)	4	58.9 (25.6)	10	0.59^b^
Activity Impairment, %	50.0 (26.5)	27	7.0 (61.4)	7	52.4 (26.3)	34	0.22^b^
Walk Independently							
Absenteeism, %	4.4 (8.1)	6	6.8 (15.1)	12	6.0 (12.9)	18	0.91^b^
Presenteeism, %	17.5 (22.5)	8	37.3 (25.5)	15	30.4 (25.8%)	23	0.07
Overall Impairment, %	25.5 (26.5)	6	35.5 (28.1)	12	32.2 (27.2)	18	0.40^b^
Activity Impairment, %	41.2 (24.2)	25	55.0 (22.3)	26	48.2 (24.1)	51	0.02*^,b^

^a^ Significance testing between work productivity of a caregiver and an affected adult

*Statistically significant at p < 0.05

^b^ Mann-Whitney test used to test for significance

Among caregivers, the greatest levels of activity impairment were experienced among those caring for affected individuals on permanent ventilation, 83.1%). While caregivers of affected individuals that can walk independently have the lowest levels of productivity loss, as one would expect, across all the functional milestone groups, all caregivers are still experiencing a 25.5% loss of overall work productivity (overall impairment) and 41.2% loss of non-work activity impairment.

A linear regression analysis (Table 5) was completed to determine if work productivity was impacted by the age of the affected individual. Absenteeism was inversely related to the age of the affected individual, with increasing levels of absenteeism with decreasing age of the affected individual. Age of the affected individual did not explain variance in presenteeism, overall impairment and activity impairment.

**Table 5:** Absenteeism by age of affected individual, in months

	Unstandardized Coefficients	Standardized Coefficients
Age During Survey, in months	−0.03 (0.01)***	−0.29***
Constant	19.36 (2.90)	

Observations: 131.

***p < 0.01

### PROMIS Fatigue SF

**Fig. 3:**
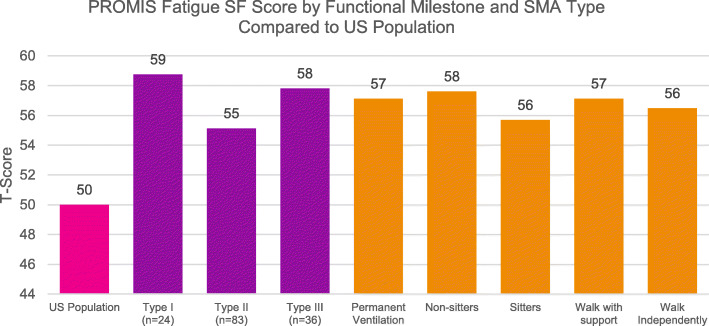
PROMIS Fatigue SF Score by Functional Milestone and SMA Type Compared to US Population

For assessment of fatigue, parents of affected children ages 5–17 completed the PROMIS Fatigue SF assessment tool and results were stratified by functional milestone and by SMA type. Higher scores on this measurement indicate greater fatigue. Raw scores were rescaled to standardized T-scores to allow for relevant comparison among sub-groups and to the overall US population (Fig. 3).

The adjusted T-scores ranged from 55.69–57.61 when assessing PROMIS scores by functional status. All the scores were worse than the average general population score of 50, but there did not appear to be a trend of scores increasing or decreasing by SMA type or functional status (Table 6).

**Table 6:** PROMIS Fatigue SF T-Scores by Functional Milestone and SMA Type

Functional Status	Type I	Type II	Type III
Permanent Ventilation			
n	17	2	0
Mean (SD)	56.6 (10.2)	62 (11.3)	
Range	(42–71)	(54–70)	
Non-sitters			
n	2	16	0
Mean (SD)	56 (8.5)	57.8 (10.2)	
Range	(50–62)	(39–71)	
Sitters			
n	5	55	7
Mean (SD)	67.2 (9.5)	54.3 (9.0)	58.4 (5.4)
Range	(54–80)	(34–74)	(48–65)
Walk with Support			
n	0	7	9
Mean (SD)		56.4 (8.9)	57.7 (7.4)
Range		(39–63)	(47–67)
Walk Independently			
n	0	3	20
Mean (SD)		48.7 (8.4)	57.7 (9.4)
Range		(39–54)	(34–73)

-Only for unique individuals

A factor analysis evaluated the results from each question to determine if there was a statistically significant difference between the average value of the answer and the independent categories of either SMA type or the functional status (Sup. Table 1).

For example, in question 1, the average response answer was 2.71 for type I, 2.34 for type II, and 2.78 for type III and there was no statistically significant difference between these averages. More specifically, the question elements “My child felt weak” and “My child was so tired it was hard for him/her to pay attention” showed a statistically significant (or nearly statistically significant) relationship to SMA type and functional status. However, weakness is a hallmark characteristic of SMA, so these questions may not be reflective of the fatigue an affected individual typically experiences. The other question elements, such as “My child was too tired to do sports or exercise” and “Being tired make it hard for my child to play or go out with friends as much as he/she would like” did not have any statistical significant relationship to SMA type or functional status. Being that the majority of individuals with SMA are non-ambulatory, a question related to individuals being involved in traditional sports, may be deemed non-applicable. This further highlights the need for more SMA-specific outcomes that includes items of most relevance by function.

## Discussion

### Overall burden

The data here presented examined various PROMs that assess specific aspects of the patient experience that might be more sensitive in capturing subtle, but meaningful changes with drug therapy administration and thereby benchmark the progression of SMA, and other aspects of disease impact such as quality of life, work productivity, and fatigue. Leveraging its annual online Community Update Survey [20], in 2019 Cure SMA engaged respondents in completing three commonly used PROMs: the Health Utilities Index (HUI), Work Productivity and Activity Impairment (WPAI) and the Patient Reported Outcomes Measurement Information System Fatigue Short Form (PROMIS Fatigue SF). These instruments were chosen to quantitatively measure various quality of life aspects impacted by SMA as studied qualitatively through previous studies in an SMA population [7, 29, 33].

Results demonstrate a highly rated burden of SMA across all three assessments that were measured, supporting findings from previous qualitative studies that assessed the burden of SMA across phenotypes [7, 11, 13]. As measured by the HUI, the quality of life scores fell under the category of ‘severe disability;’ work productivity lost due to having SMA or caring for someone with SMA was also significant; and the fatigue levels of children affected with SMA was greater than that of the general population regardless of type. Nonetheless, given the validation (age range) and parameters (mobility, emotional, etc.) assessed in each scale, we anticipate that different scales may be appropriate for different SMA subtypes and ages. By conducting similar assessments with these and other measures, we can leverage the results of this study as a baseline for understanding the burden of SMA in our community in the future.

### Strengths and limitations of HUI3 for SMA populations

The results from the HUI3 varied by SMA type and functional status. Unlike the other HUI systems, the HUI3 measures a person’s ability to walk and their dexterity which are key manifestations of SMA and vary across subtypes. As shown by the individual HUI attribute scores, there was a statistically significant difference between SMA types when evaluating the scores of ambulation and dexterity. There was also a statistically significant difference between speech attribute scores across the SMA types with type I showing the lowest speech scores representing severe disability. Although future analyses of evaluating HUI attribute scores by SMA type should adjust for age of the affected individual to determine if differences in speech are due to SMA type, age, or a combination of both. Additionally, the HUI3 measures someone’s ability to perform daily activities, such as dressing, which is a common activity that patients with SMA would like to improve or maintain [14, 33]. Assessing the HUI3 before and after treatment would allow the measurement of progression in the SMA community. However, for those who may never gain motor milestones, the HUI3 may not be a good measurement to detect any changes over time. Moreover, not all the attributes assessed by the HUI3 may be relevant for any of the SMA population. For example, the HUI3 assesses quality of life through vision and hearing attributes, which are not relevant to the manifestation of SMA across types, and the high attribute scores as shown in this analysis, demonstrate this.

### Strengths and limitations of WPAI for SMA populations

Unlike the HUI and the PROMIS, the WPAI can measure both the quality of life from the caregiver or the affected adult perspective. Interestingly, there was not a statistically significant difference in the WPAI sub-scores for caregivers and affected adults in the majority of the scores suggesting that SMA affects the work productivity the same for both the caregiver and the affected individual. However, the absenteeism score of the WPAI was influenced by the age of the affected individual with higher absenteeism scores among caregivers for younger affected individuals. Moreover, the WPAI results across functional status and SMA type show that the WPAI is sensitive to show the differences in severity across the SMA population. However, most of the affected adults that completed a survey or had a survey completed on their behalf were attending school either full or part-time. Therefore, the work productivity lost may be underrepresented in the total SMA population. A scale that redefines productivity as a student will be beneficial to assessing the entire picture of productivity lost. Lastly, it is not uncommon for families affected with SMA to hire professionals to assist with providing care to the affected individual. A limitation of this study was the demographics and employment status of the caregiver was not captured. It would be of interest to control for differences among caregivers to help parse out the differences in work productivity lost among families.

### Strengths and limitations of PROMIS fatigue for SMA

The PROMIS Fatigue SF is a short 10 question questionnaire that requires little time to complete and offers a parent proxy version that allows fatigue assessment in children. The results from the Community Update Survey show higher levels of reported fatigue among those with SMA when compared to the general population. However, when converting the PROMIS Fatigue scores into T-scores, the T-scores among those with SMA were less than one standard deviation above the general population mean. Additional analyses analysis suggested that some of these questions are not appropriate for an SMA population and/or cognitive and/or mental factors may be more relevant over physiological factors. Similarly, a recent study by Dunaway Young, et al. assessed the relationship of perceived fatigue to fatigability, function and quality of life in SMA and found that perceived fatigue did not correlate with fatigability or function [30].

### Opportunities for future efforts

It will become increasingly important to measure qualitatively and quantitatively, other aspects of physical/emotional/psychosocial functioning; including but not limited to quality of life (such as activities of daily living, levels of achieved independence, etc.) and fatigue through patient reported outcomes for ongoing reimbursement of currently approved drugs. As new treatments are approved, the community’s expectations from a given therapy, will likely evolve. As such, our ability to effectively capture clinically meaningful changes across current functional abilities and through various outcomes (motor function, respiratory function, HRQL, SMA-specific PROMs, etc.) must also evolve to ensure we are able to effectively capture meaningful change across the SMA community as therapeutic options evolve.

## Conclusions

The 2019 Community Update Survey dataset provides an important benchmark from which to begin assessing year-over-year change in HRQoL for affected individuals and their caregivers. Cure SMA will conduct follow up annual surveys using the WPAI and HUI instruments to evaluate the impact that new therapies are making on the overall experience of affected individuals and their families. It is anticipated that these future survey activities will also add in other HRQoL measurements (including the EQ-5D [34] and the Fatigue Impact Scale [35]) to broaden the picture of SMA impact among the community, evaluate which tools are most sensitive to each subtype of the diverse SMA population, can assess treatment affects and determine the health utility among a large sample of affected individuals with SMA.

Ultimately, we anticipate learning through this process, that different instruments will be more appropriate for assessing HRQoL within SMA, and among the various SMA subpopulations, ages of affected individuals and functional milestone status.

## Supplementary information

**Additional file 1.**

## Data Availability

The data collected and analyzed during the current study is generated and owned by Cure SMA and not publicly available.
