# Supplementary material for: Quality of life data for individuals affected by spinal muscular atrophy: a baseline dataset from the Cure SMA Community Update Survey
Source: Orphanet J Rare Dis. 2020 Aug 24;15:217. doi: 10.1186/s13023-020-01498-2 (PMC7447573; doi:10.1186/s13023-020-01498-2)
Supplement: Supplementary file 1 — Additional file 1. [file 13023_2020_1498_MOESM1_ESM.docx]

Supplemental:

| **Supplemental Table 1: PROMIS Fatigue SF Questionnaire Factor Analysis** | | | | | | | | | | | | | | | | | | | | | |
| --- | --- | --- | --- | --- | --- | --- | --- | --- | --- | --- | --- | --- | --- | --- | --- | --- | --- | --- | --- | --- | --- |
|  | | **Question 1** | | **Question 2** | | **Question 3** | | **Question 4** | | **Question 5** | | **Question 6** | | **Question 7** | | **Question 8** | | **Question 9** | | **Question 10** | |
| SMA Type | | Mean | p value | Mean | p value | Mean | p value | Mean | p value | Mean | p value | Mean | p value | Mean | p value | Mean | p value | Mean | p value | Mean | p value |
|  | Type I | 2.71 | 0.09 | 3.79 | 0.0056 | 3 | 0.09 | 2.75 | 0.25 | 2.46 | 0.578 | 2.26 | 0.6542 | 2.54 | 0.07 | 2.92 | 0.0979 | 2.54 | 0.0087 | 2.13 | 0.07 |
|  | Type II | 2.34 |  | 2.99 |  | 2.65 |  | 2.48 |  | 2.54 |  | 2.09 |  | 2.11 |  | 2.52 |  | 2.16 |  | 2.06 |  |
|  | Type III | 2.78 |  | 3.17 |  | 3.08 |  | 2.28 |  | 2.33 |  | 2.19 |  | 2.06 |  | 3 |  | 2.78 |  | 2.47 |  |
| Functional Status | |  |  |  |  |  |  |  |  |  |  |  |  |  |  |  |  |  |  |  |  |
|  | Permanent Ventilation | 2.58 | 0.69 | 3.74 | 0.15 | 2.79 | 0.86 | 2.63 | 0.52 | 2.37 | 0.54 | 2.17 | 0.99 | 2.37 | 0.14 | 2.63 | 0.9659 | 2.37 | 0.35 | 1.95 | 0.4 |
|  | Non sitters | 2.56 |  | 3.06 |  | 2.78 |  | 2.72 |  | 2.67 |  | 2.22 |  | 2.44 |  | 2.83 |  | 2.56 |  | 2.33 |  |
|  | Sitters | 2.37 |  | 3.14 |  | 2.75 |  | 2.48 |  | 2.57 |  | 2.11 |  | 2.05 |  | 2.66 |  | 2.19 |  | 2.09 |  |
|  | Walk with support | 2.63 |  | 3.13 |  | 2.88 |  | 2.38 |  | 2.38 |  | 2.13 |  | 2.44 |  | 2.69 |  | 2.63 |  | 2.31 |  |
|  | Walk independently | 2.74 |  | 2.91 |  | 3.04 |  | 2.17 |  | 2.22 |  | 2.17 |  | 1.96 |  | 2.83 |  | 2.61 |  | 2.39 |  |
